# Biological sex-related differences in whole-body coordination during standing turns in healthy young adults

**DOI:** 10.1038/s41598-023-49201-2

**Published:** 2023-12-13

**Authors:** Fuengfa Khobkhun, Jenjira Thanakamchokchai

**Affiliations:** Parkinson Movement and Research Collaboration Laboratory, Faculty of Physical Therapy, Mahidol University, Salaya, 73170 Nakhon Pathom Thailand

**Keywords:** Public health, Geriatrics

## Abstract

Biological sexes (male and female) have been reported to influence postural control and balance due to differences in musculoskeletal structures, hormonal factors, and neuromuscular control. These factors can contribute to the turning performance, potentially leading to an increased incidence of falls, particularly during turning. Therefore, this study aimed to explore the whole-body coordination and stepping characteristics and during standing turns in healthy adults to determine the effects of biological sex and turn speed. Fifty participants (25 males and 25 females) completed 180° standing turns on level ground. Inertial Measurement Units (XSENS) were used to measure whole-body movement turning kinematics and stepping characteristics. Moreover, clinical outcome of dynamic balance was measured by the Timed Up and Go (TUG). Participants were randomly tasked to turn at three speeds; fast, moderate, or slow to the left and right sides. Mann–Whitney *U* tests were used to compare the independent variables between male and females, and Friedman tests with Dunn’s tests for pairwise comparisons were used to compare between the three turning speeds within the two groups. The results demonstrated that significant differences were seen between males and females during fast turning for the leading foot onset (p = 0.048) and in the slow speed for the total step (p = 0.033), showing that these were greater in female with an increase in turn speed. In addition, significant differences were seen only in the males when comparing different speeds in the trailing foot onset latency (p = 0.035), step size (p = 0.009), and total number of steps (p = 0.002), while in the females a significant difference was found in peak head yaw velocity between fast and slow turn speeds, and moderate and slow turn speeds. Finally, there was no significant difference in TUG between groups. Therefore, these findings show differences between biological sexes in the response to whole-body coordination and step characteristics, indicating that females tend to have more changes in stepping characteristics compared to males due to differences in turning speed. This can affect their balance and stability. However, the differences in biological sex did not impact the dynamic balance and fall risk due to the lack of a significant difference shown by TUG between males and females.

## Introduction

Turning is a fundamental but complex component of locomotion which is controlled by the central nervous system and requires the coordination of whole-body reorientation towards a new travel direction and the maintenance of whole-body stability in the medial–lateral plane during a turn^1^. Studies focusing on both on-the-spot and steering turns, i.e. changing the direction of walking, have shown a common stereotypical movement sequence^1–4^. Turning is a top-down sequence of movement, initiated by saccadic eye movements to shift the gaze in the direction of travel, followed by the rotation of the head, trunk, and pelvis, and finally, the stepping movements of the feet^4–9^. These apparently straightforward movements necessitate a complex interplay among multiple muscle groups and joint actions to both maintain balance and execute precise turns.

In addition, it has been found that biological sex has an influence on postural control and balance^10,11^. Research suggests that there are inherent differences between males and females in terms of musculoskeletal structure, hormonal factors, and neuromuscular control, which can contribute to variations in postural control and balance abilities^11–24^. For musculoskeletal structure, studies have found that females generally have a lower centre of gravity and wider pelvis compared to males^11,14,15^. This anatomical difference can affect the distribution of body mass and alter the biomechanics of postural control. For example, the wider pelvis leads to a decrease in the effective mechanical advantage of the hip abductor muscles, and the maintenance of pelvic stability during a single leg support activity such as running or walking which require more muscle force and more metabolic energy to control the stability^11,13–15^. Additionally, hormonal factors such as oestrogen and progesterone levels can impact ligament laxity and joint stability, potentially influencing balance control^13,15,16^. A previous study indicated a low level of testosterone has been related to a resultant increase in fall risk related to a reduction in muscle mass, strength and physical performance^17^. Accordingly, an increase in the level of testosterone helps to improve maximal voluntary strength and power in male^18^. Furthermore, differences in neuromuscular control between the biological sexes have been observed^19,20^. Females tend to exhibit greater reliance on sensory systems, such as visual and proprioceptive cues, for maintaining balance, whereas males may rely more on muscular strength and power^20–22^. These variations in sensory and motor strategies can affect the ability to maintain stability and adapt to different postural challenges. It is important to note that while there are general trends, individual variations within each biological sex can be significant with factors such as age, physical fitness, and training also influencing postural control and balance abilities. In addition, a previous study has shown that females are less physically active than males^23^. It has been demonstrated that lower levels of physical activity can have an impact on postural control, balance, muscle strength, and coordination, leading to an increase in the propagational force of plantar shear during static and dynamic tasks. Furthermore, ankle muscle activity necessitates greater co-contraction for control^24^. Therefore, the level of physical activity can influence turning characteristics, which are crucial for executing turns effectively. Also, a slower Timed Up and Go (TUG) test was found to be associated with advanced age and being female, with age being the primary factor influencing the results^25^. Thus, investigating differences between the biological sexes is important, as it not only provides insights into the fundamental principles of turning movement but also carries practical implications for various fields, including physical therapy, sports science, and ergonomics.

In studies involving healthy adults of both biological sexes, with a focus on functional performance, it has been demonstrated that the male advantage regarding throwing accuracy remains consistent, independent of various paper-and-pencil spatial tasks^26^. This is in addition to mental rotation, a task in which males tend to outperform females^27^. Initially, practice was considered a potential factor contributing to gender differences in throwing accuracy^28^, but subsequent analyses indicated that this difference persists even when taking into account the influence of the sports history of the individual. Extensive research has explored the biomechanics of turning movements in healthy adults^1,4,29,30^ and individuals with neurological conditions^8,9,31–34^, the finding of which have added weight to the evidence concerning turning differences in the biological sexes. Also, previous studies have shown that gender influences postural stability, balance, and gait speed; specifically, males walk faster than females, a finding often attributed to longer step and stride length in male^35,36^. There remains a notable gap in our understanding into potential biological sex-based differences in whole-body coordination during standing turns. In addition, there have been no reports on the differences between the biological sexes regarding turning characteristics, and a comprehensive understanding of the impact of biological sex on postural control and balance is lacking. This study aims to address this research gap by conducting a comprehensive analysis of whole-body coordination during standing turns in healthy adult males and females observing the corresponding effects on body coordination and stepping characteristics and their responses to turning speed. Specifically, this study aims to explore turning characteristics that are associated with dynamic balance and are related to the risk of falls. These findings can then be utilized to develop fall prevention programs for various populations. We hypothesized that differences in whole-body coordination, stepping characteristics, and responses to turning speeds would exist between the biological sexes. We also postulated that females might take more time, have less angular separations, and exhibit more step control than males during turning. The findings of this study will contribute to our broader understanding of how the human body adapts to different motor tasks, especially in tasks related to turning and may shed light on the development of more targeted interventions for individuals with specific coordination challenges and also related to biological sex.

## Methods

### Participant preparation

All participants were recruited from the local community of Salaya, Nakhon Pathom, Thailand. In addition, the following inclusion criteria were considered; aged between 18 and 75 of both biological sexes (male and female), able to follow commands and instructions, able to walk independently without any assistive device, have sufficient cognitive ability to understand the questionnaire and follow commands (mini-Thai mental state examination ≥ 24/30)^37^, and no clinical diagnosis of diseases or symptoms that could influence the test performance such as arthritis or severe leg pain. The following exclusion criteria were used; comorbidity with severe systemic illness, severe sign and symptoms of musculoskeletal problems which influence test performance. All participants were asked to read the participant information sheet and signed an informed consent form prior to data collection. The study was approved by the Mahidol University Institutional Review Board (COA No. MU-CIRB 2022/088.1508) which was carried out fully in accordance the ethical standard guidelines of the Declaration of Helsinki.

### Turning protocol and data collection

Demographic data was recorded, including weight, height, body mass index (BMI), leg length and underlying disease. In addition, participants were given self-administered survey questionnaires that were originally developed in English using the "Global Physical Activity Questionnaire"^38^ as a foundation and then translated into Thai using the method described by Kuder-Richardson Formula 20^39^. The reliability of the questionnaire was established through a test–retest analysis (0.751, p < 0.001), and it also demonstrated strong internal consistency (alpha coefficient = 0.727) among healthy adults^39^.

### Turning kinematics and stepping characteristics assessments

All participants turning kinematics were measured using XSENS DOT Inertial Measurement Units (XSENS Technologies B.V., P.O. Box 559, 7500 AN Enschede, the Netherlands) which was validated and used to measure whole body movement turning kinematics and stepping characteristics at a sampling frequency of 60 Hz^40^. IMUs were strapped firmly to the body segments including; the centre of the head, middle thorax, pelvis, both thighs, both shanks, and the centre of the left and right feet.

**Figure 1:**
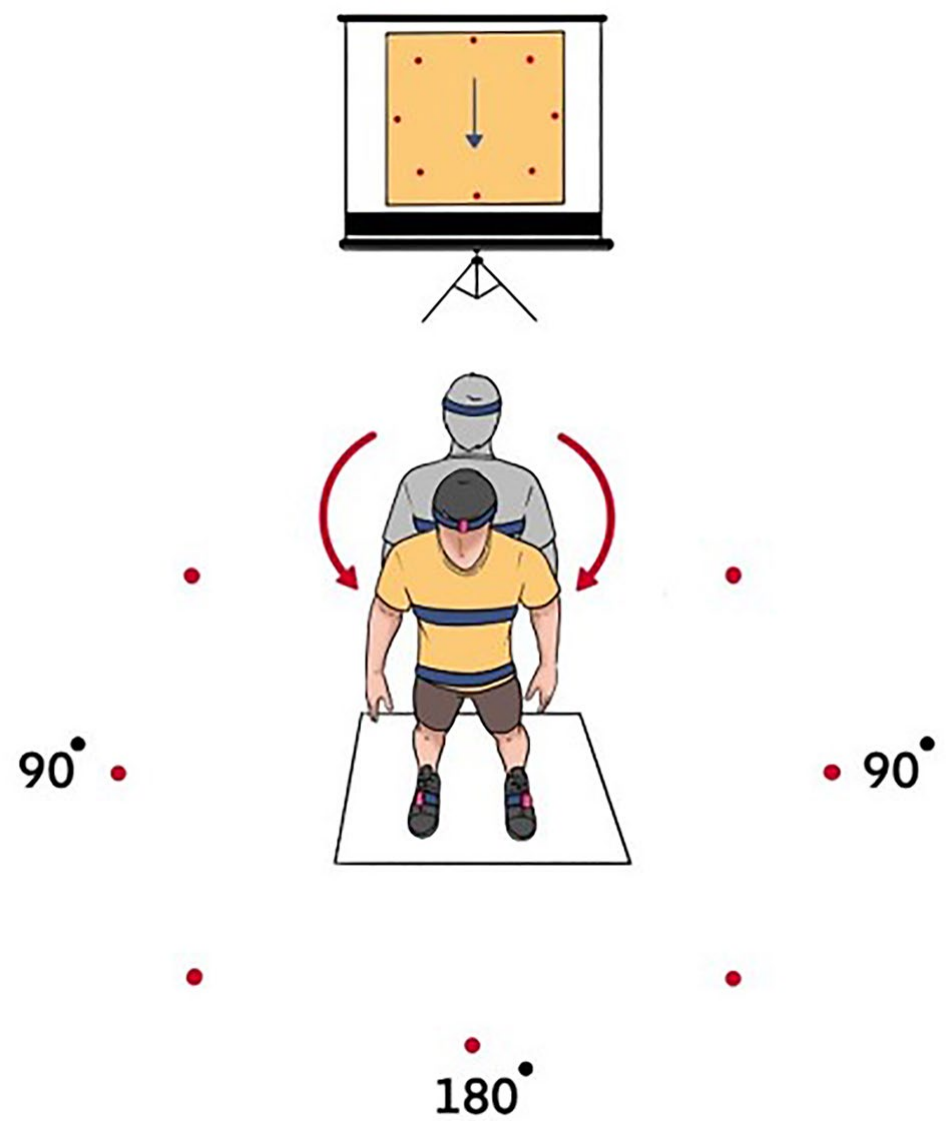
A representation of the video screen and participants standing position showing the different directions and speeds of turn.

Participants were asked to stand facing a laptop screen and were asked to perform 180° standing turns at three different speeds: fast (1.5 s), moderate (2 s), and slow (3 s). The timing of the completion for these speeds was indicated by the audio signals which were produced by LABVIEW and have been previously used to study turning speeds during 180° turns^3,29^. Prior to each trial, a video was shown of an animation demonstrating the turn by a programme in LABVIEW which showed a representation of the turn which the participants were asked to imitate focussing on the direction and amplitude of the animated clock arm as accurately as possible, Fig. 1. Therefore, six trials in total were recorded for three turning speeds and two directions for each speed. The order of the speeds and directions was randomised for each participant.

### Turning speed difficulty

After completing the data collection, each participant was asked about the difficulty of each turn speed using the question, "Which turning speed do you think is the most difficult?" And “which turning speed do you think is the easiest?”. All responses were collected using a Google form survey.

### Timed up and go (TUG) test

The TUG test is a clinical outcome measure used to assess dynamic balance and mobility, which includes a turning subtask. The TUG test measures the time that a participant needs to stand up from a chair, walk 3 m, turn, walk back to the chair, and sit back down with the normal speed^16,23^. The TUG test was administered to the participants as the final component of data collection.

### Data processing

Euler angles from the XSENS DOTS systems were exported to determine the angular displacement of the head, thorax, pelvis and left and right feet in the global reference frame. All dependent variables for each segment including segment onset latency, intersegmental coordination (head on pelvis and head on thorax), and individual stepping characteristics including step duration, step size, number of steps and step frequency were extracted using a MATLAB (R2023a) based on a previously published methodology^3,29^.

### Statistical analysis

The number of participants needed for this study was determined based on head onset latency from a previous study that used a similar approach^29^. To ensure statistical power of 90% and a significance level of 5%, it was calculated that a sample size of 40 participants was needed using Gpower software, with 20 males and 20 females in each group. Considering a potential dropout rate of 10%, 50 participants, with 25 males and 25 females in each group, were recruited for this study.

The data distribution was tested using Shapiro–Wilk tests, which indicated that the data did not adhere to the pattern a normal distribution. Therefore, non-parametric testing was suitable for analysis. Demographic data, TUG test, strategy used for turning 180° and the speed of turning difficulty were performed using the Mann–Whitney *U* test for non-normality continuous data, and physical activity factor comparisons between male and female groups were performed using the Chi-square test and Fisher’s exact test for categorical data. Friedman tests with Dunn’s pairwise comparisons tests were used to explore any differences between the three turning speeds (fast, normal, and slow). All statistical analyses were performed in IBM SPSS Statistics 24 (IBM Corporation, Armonk, NY) and the significance level was set at p < 0.05.

## Results

Fifty-eight participants were recruited, however, only 50 (25 males and 25 females with matched ages) of these completed the protocol. No significant differences were seen between the biological sexes for; age, marital status, education levels, underlying disease, and physical activity. However, there was a significant difference between the biological sexes with regards to weight, height, and Body Mass Index (BMI) (Table 1).

**Table 1:** Demographic data and physical activity comparisons of participants between male and female groups.

Factors	Male (n = 25)	Female (n = 25)	p-value^#^
Part 1			
Age (years)	21.0 (20.5, 42.0)	22.0 (19.0, 59.5)	0.953
Weight (kg)	67.0 (63.0, 76.5)	54.0 (50.0, 61.5)	< 0.001*
Height (cm)	171.0 (167.5, 175.0)	160.0 (157.0, 165.3)	< 0.001*
Body Mass Index (kg/m^2^)	22.6 (21.8, 25.2)	20.3 (19.0, 23.8)	0.011*
Marital status (n, %)			1.000
Married	6 (24%)	6 (24%)	
Single	19 (76%)	19 (76%)	
Education levels (n, %)			0.078
Under Bachelor degree	1 (4%)	3 (12%)	
Bachelor degree	20 (80%)	22 (88%)	
Higher than Bachelor degree	4 (16%)	0 (0%)	
Underlying disease			0.123
No	15 (60%)	20 (80%)	
Yes	10 (40%)	5 (20%)	
Part 2			
Activity at work			
Does your work involve vigorous-intensity activity that causes large increases in breathing or heart rate like [carrying or lifting heavy loads, digging or construction work] for at least 10 min continuously?			1.000
Yes	2 (8%)	1 (4%)	
No	23 (92%)	24 (96%)	
In a typical week, on how many days do you do vigorous-intensity activities as part of your work?	3.5	1.0	N/A
How much time do you spend doing vigorous-intensity activities at work on a typical day?	90	30	N/A
Does your work involve moderate-intensity activity that causes small increases in breathing or heart rate such as brisk walking [or carrying light loads] for at least 10 min continuously?			1.000
Yes	10 (40%)	11 (44%)	
No	15 (60%)	14 (56%)	
In a typical week, on how many days do you do moderate-intensity activities as part of your work?	3 (2, 5)	3 (3, 6)	0.314
How much time do you spend doing moderate-intensity activities at work on a typical day?	60 (35, 60)	30 (20, 60)	0.295
Travel to and from places			
Do you walk or use a bicycle (pedal cycle) for at least 10 min continuously to get to and from places?			1.000
Yes	21 (84%)	21 (84%)	
No	4 (16%)	4 (16%)	
In a typical week, on how many days do you walk or bicycle for at least 10 min continuously to get to and from places?	5 (3, 5)	5 (4, 7)	0.307
How much time do you spend walking or bicycling for travel on a typical day?	30 (20, 60)	25 (15, 45)	0.258
Recreational activities			
Do you do any vigorous-intensity sports, fitness or recreational (leisure) activities that cause large increases in breathing or heart rate like [running or football] for at least 10 min continuously?			0.254
Yes	13 (52%)	9 (36%)	
No	12 (48%)	16 (64%)	
In a typical week, on how many days do you do vigorous-intensity sports, fitness or recreational (leisure) activities	3 (3.0, 4.5)	3.0 (1.0, 3.5)	0.471
How much time do you spend doing vigorous-intensity sports, fitness or recreational activities on a typical day?	120 (60, 120)	30 (30, 60)	0.009*
Do you do any moderate-intensity sports, fitness or recreational (leisure) activities that causes a small increase in breathing or heart rate such as brisk walking, (cycling, swimming, volleyball) for at least 10 min continuously?			0.239
Yes	14 (56%)	18 (72%)	
No	11 (44%)	7 (28%)	
In a typical week, on how many days do you do moderate-intensity sports, fitness or recreational (leisure) activities?	3.0 (1.5, 5.0)	3.5 (2.8, 6.3)	0.258
How much time do you spend doing moderate-intensity sports, fitness or recreational (leisure) activities on a typical day?	60 (40, 60)	30 (25, 60)	0.175
Sedentary behaviour			
How much time do you usually spend sitting or reclining on a typical day?	8.0 (3.5, 16.0)	8.0 (5.0, 13.0)	0.892

^#^Mann–Whitney *U* test for non-normality continuous data, chi-square test and Fisher’s exact test for categorical data.

*Statistical significance.

For the turning kinematics variables, the Mann–Whitney *U* test only showed statistical differences between the male and female groups in the fast speed for the leading foot onset (p = 0.048, effect size = 0.524) and in the slow speed for the total step (p = 0.033, effect size = 0.630) (Table 2). When considering speeds the Friedman test showed a significant main effect within the male group for head (p < 0.001), thorax (p = 0.011), pelvis (p < 0.001), leading foot (p < 0.001) and trailing foot yaw onset latencies (p = 0.035), peak head on pelvis (p = 0.005), step size (p = 0.009), total step (p = 0.002) and step duration (p < 0.001) (Table 3). In addition, Dunn’s test for pairwise comparisons showed a significant difference among speeds within the male group between fast and moderate speeds (p = 0.009, effect size = 0.678) and fast and slow speeds (p < 0.001, effect size = 1.033) for the head onset latency, fast and slow speeds for the thorax onset latency (p = 0.014, effect size = 1.006), fast and moderate speeds (p = 0.003, effect size = 1.126) and fast and slow speeds (p = 0.001, effect size = 1.133) for the pelvis onset latency, fast and moderate speeds for the leading foot (p = 0.001, effect size = 1.016) and the trailing foot (p = 0.033, effect size = 0.619), fast and slow speed for peak head on pelvis (p = 0.003, effect size = 0.545), step size (p = 0.009, effect size = 1.117) and total step (p = 0.009, effect size = 0.788), showing that these were greater with an increase in turn speed. For step duration, Dunn’s test pairwise comparisons found a significant difference between fast and moderate speeds (p = 0.001, effect size = 0.806), fast and slow speeds (p < 0.001, effect size = 0.948) and moderate and slow speeds (p = 0.001, effect size = 0.831), showing that these were greater with an increase in turn speed (Table 3).

**Table 2:** Comparison of turning kinematics between male and female groups by using Mann–Whitney *U* test.

Variables	Male (n = 25)	Female (n = 25)	p-value^#^	Effect size
	Median (Q1, Q3)	Median (Q1, Q3)		
Fast speed				
Head yaw onset (s)	0.328 (0.292, 0.373)	0.323 (0.223, 0.371)	0.634	0.110
Thorax yaw onset (s)	0.366 (0.333, 0.386)	0.340 (0.292, 0.377)	0.187	0.405
Pelvis yaw onset (s)	0.430 (0.399, 0.472)	0.393 (0.353, 0.478)	0.684	0.051
Leading foot onset (s)	0.459 (0.428, 0.528)	0.514 (0.460, 0.589)	0.048*	0.524
Trailing foot onset (s)	0.540 (0.470, 0.720)	0.705 (0.480, 0.791)	0.133	0.462
Peak head yaw velocity (°s^−1^)	295.378 (262.264, 340.086)	293.359 (267.664, 340.967)	0.900	0.095
Peak head on thorax (°)	26.947 (16.765, 39.133)	24.273 (13.180, 32.211)	0.233	0.296
Peak head on pelvis (°)	23.446 (17.508, 40.243)	21.966 (14.584, 33.982)	0.541	0.163
Step size (°)	121.582 (91.730, 144.590)	111.517 (91.130, 123.615)	0.648	0.161
Total step (n)	2.5 (2.0, 3.0)	2.5 (2.5, 3.0)	0.659	0.031
Step duration (s)	1.656 (1.623, 1.697)	1.652 (1.626, 1.717)	0.509	0.278
Step frequency (Hz)	1.786 (1.216, 2.484)	1.591 (1.152, 2.469)	0.567	0.223
Moderate speed				
Head yaw onset (s)	0.390 (0.330, 0.470)	0.415 (0.231, 0.480)	0.907	0.072
Thorax yaw onset (s)	0.437 (0.392, 0.500)	0.433 (0.338, 0.518)	0.930	0.189
Pelvis yaw onset (s)	0.511 (0.483, 0.529)	0.520 (0.460, 0.585)	0.497	0.344
Leading foot onset (s)	0.600 (0.497, 0.704)	0.580 (0.471, 0.740)	0.961	0.163
Trailing foot onset (s)	0.630 (0.518, 0.975)	0.537 (0.444, 0.911)	0.190	0.266
Peak head yaw velocity (°s^−1^)	295.902 (183.773, 372.659)	229.500 (195.342, 323.690)	0.377	0.400
Peak head on thorax (°)	21.997 (17.084, 47.273)	21.386 (13.293, 31.575)	0.377	0.368
Peak head on pelvis (°)	19.281 (11.604, 29.774)	16.927 (8.432, 23.793)	0.594	0.057
Step size (°)	92.067 (81.930, 117.786)	104.577 (89.493, 125.182)	0.367	0.291
Total step (n)	3.0 (2.5, 3.0)	2.5 (2.5, 3.5)	0.872	0.001
Step duration (s)	2.148 (2.107, 2.279)	2.179 (2.138, 2.233)	0.621	0.272
Step frequency (Hz)	2.191 (1.262, 2.751)	1.908 (1.278, 2.667)	0.764	0.147
Slow speed				
Head yaw onset (s)	0.420 (0.370, 0.456)	0.449 (0.360, 0.508)	0.426	0.187
Thorax yaw onset (s)	0.459 (0.404, 0.472)	0.450 (0.416, 0.570)	0.399	0.398
Pelvis yaw onset (s)	0.523 (0.470, 0.552)	0.510 (0.495, 0.578)	0.839	0.263
Leading foot onset (s)	0.541 (0.513, 0.692)	0.680 (0.510, 0.822)	0.260	0.450
Trailing foot onset (s)	0.593 (0.555, 0.948)	0.750 (0.513, 0.931)	0.869	0.188
Peak head yaw velocity (°s^−1^)	265.249 (144.903, 331.529)	183.430 (149.310, 314.447)	0.705	0.303
Peak head on thorax (°)	20.580 (10.203, 35.991)	19.681 (7.450, 36.150)	0.691	0.010
Peak head on pelvis (°)	13.779 (10.615, 24.123)	10.599 (6.208, 20.756)	0.097	0.341
Step size (°)	91.249 (86.670, 94.043)	111.362 (82.585, 123.550)	0.079	0.601
Total step (n)	3.0 (3.0, 3.5)	3.0 (3.0, 3.0)	0.033*	0.630
Step duration (s)	3.131 (3.061, 3.204)	3.181 (3.108, 3.225)	0.204	0.486
Step frequency (Hz)	2.829 (1.388, 3.182)	2.025 (1.068, 3.057)	0.138	0.294

*Statistical significance.

*Q1 First quartile, *Q3* Third quartile.

*F Fast speed, *M* Moderate speed, *S* Slow speed.

(°) - degree and (°s^−1^) - degrees per second.

**Table 3:** Comparison within group among three speeds by using Friedman test and pairwise comparison by Dunn’ s test.

Groups and variables	Fast speed (F) Median (Q1, Q3)	Moderate speed (M) Median (Q1, Q3)	Slow speed (S)	p-value^#^	p-value^#^ (effect size)		
			Median (Q1, Q3)		F vs M	F vs S	M vs S
Male							
Head yaw onset (s)	0.328 (0.292, 0.373)	0.390 (0.330, 0.470)	0.420 (0.370, 0.456)	< 0.001*	0.009* (0.678)	< 0.001* (1.033)	1.000 (0.252)
Thorax yaw onset (s)	0.366 (0.333, 0.386)	0.437 (0.392, 0.500)	0.459 (0.404, 0.472)	0.011*	0.071 (0.721)	0.014* (1.006)	1.000 (0.198)
Pelvis yaw onset (s)	0.430 (0.399, 0.472)	0.511 (0.483, 0.529)	0.523 (0.470, 0.552)	< 0.001*	0.003* (1.126)	0.001* (1.133)	1.000 (0.167)
Leading foot onset (s)	0.459 (0.428, 0.528)	0.600 (0.497, 0.704)	0.541 (0.513, 0.692)	< 0.001*	0.001* (1.016)	0.006 (1.016)	1.000 (0.015)
Trailing foot onset (s)	0.540 (0.470, 0.720)	0.630 (0.518, 0.975)	0.593 (0.555, 0.948)	0.035*	0.033* (0.619)	0.269 (0.554)	1.000 (0.153)
Peak head yaw velocity (°s^−1^)	295.378 (262.264, 340.086)	295.902 (183.773, 372.659)	265.249 (144.903, 331.529)	0.468	1.000 (0.146)	0.967 (0.324)	0.774 (0.195)
Peak head on thorax (°)	26.947 (16.765, 39.133)	21.997 (17.084, 47.273)	20.580 (10.203, 35.991)	0.340	1.000 (0.082)	0.609 (0.263)	0.609 (0.310)
Peak head on pelvis (°)	23.446 (17.508, 40.243)	19.281 (11.604, 29.774)	13.779 (10.615, 24.123)	0.005*	0.198 (0.090)	0.003* (0.545)	0.472 (0.254)
Step size (°)	121.582 (91.730, 144.590)	92.067 (81.930, 117.786)	91.249 (86.670, 94.043)	0.009*	0.102 (0.625)	0.009* (1.117)	1.000 (0.475)
Total step (n)	2.5 (2.0, 3.0)	3.0 (2.5, 3.0)	3.0 (3.0, 3.5)	0.002*	1.000 (0.150)	0.009* (0.788)	0.059 (0.723)
Step duration (s)	1.656 (1.623, 1.697)	2.148 (2.107, 2.279)	3.131 (3.061, 3.204)	< 0.001*	0.001* (0.806)	< 0.001* (0.948)	0.001* (0.831)
Step frequency (Hz)	1.786 (1.216, 2.484)	2.191 (1.262, 2.751)	2.829 (1.388, 3.182)	0.468	1.000 (0.064)	0.774 (0.394)	0.967 (0.347)
Female							
Head yaw onset (s)	0.323 (0.223, 0.371)	0.415 (0.231, 0.480)	0.449 (0.360, 0.508)	0.004*	0.071 (0.529)	0.003* (1.039)	0.967 (0.200)
Thorax yaw onset (s)	0.340 (0.292, 0.377)	0.433 (0.338, 0.518)	0.450 (0.416, 0.570)	< 0.001*	0.014* (0.830)	< 0.001* (1.591)	0.774 (0.165)
Pelvis yaw onset (s)	0.393 (0.353, 0.478)	0.520 (0.460, 0.585)	0.510 (0.495, 0.578)	< 0.001*	0.003* (0.777)	< 0.001* (0.935)	1.000 (0.055)
Leading foot onset (s)	0.514 (0.460, 0.589)	0.580 (0.471, 0.740)	0.680 (0.510, 0.822)	0.005*	0.198 (0.532)	0.003* (0.878)	0.472 (0.242)
Trailing foot onset (s)	0.705 (0.480, 0.791)	0.537 (0.444, 0.911)	0.750 (0.513, 0.931)	0.595	1.000 (0.079)	0.967 (0.232)	1.000 (0.304)
Peak head yaw velocity (°s^−1^)	293.359 (267.664, 340.967)	229.500 (195.342, 323.690)	183.430 (149.310, 314.447)	< 0.001*	0.688 (0.654)	< 0.001* (0.913)	0.022* (0.325)
Peak head on thorax (°)	24.273 (13.180, 32.211)	21.386 (13.293, 31.575)	19.681 (7.450, 36.150)	0.688	1.000 (0.032)	1.000 (0.005)	1.000 (0.023)
Peak head on pelvis (°)	21.966 (14.584, 33.982)	16.927 (8.432, 23.793)	10.599 (6.208, 20.756)	0.031*	0.688 (0.056)	0.027* (0.741)	0.472 (0.390)
Step size (°)	111.517 (91.130, 123.615)	104.577 (89.493, 125.182)	111.362 (82.585, 123.550)	0.179	0.774 (0.162)	0.198 (0.305)	1.000 (0.133)
Total step (n)	2.5 (2.5, 3.0)	2.5 (2.5, 3.5)	3.0 (3.0, 3.0)	0.097	1.000 (0.139)	0.269 (0.299)	0.867 (0.146)
Step duration (s)	1.652 (1.626, 1.717)	2.179 (2.138, 2.233)	3.181 (3.108, 3.225)	< 0.001*	0.003* (0.856)	< 0.001* (0.996)	0.001* (0.823)
Step frequency (Hz)	1.591 (1.152, 2.469)	1.908 (1.278, 2.667)	2.025 (1.068, 3.057)	0.468	0.774 (0.154)	0.967 (0.288)	1.000 (0.151)

*Statistical significance between the three speeds was calculated using Friedman test (column 4), and Dunn’s pairwise comparisons test was used to explore any differences between the three turning speeds (fast, normal, and slow) (columns 1–3 to the right).

*Q1 First quartile, *Q3* Third quartile.

*F Fast speed, *M* Moderate speed, *S* Slow speed.

(°) - degree and (°s^−1^) - degrees per second.

Within the female group, the Friedman test showed significant main effects for turn speed for head (p = 0.004), thorax (p < 0.001), pelvis (p < 0.001) and leading foot onset latencies (p = 0.005), peak head yaw velocity (p < 0.001), peak head on pelvis (p = 0.031) and step duration (p < 0.001) (Table 3). Furthermore, Dunn’s test pairwise comparisons found a significant difference among speeds within the female group between fast and moderate speeds (p = 0.003, effect size = 1.039) for the head onset latency, fast and moderate speeds (p = 0.014, effect size = 0.830) and fast and slow speeds (p < 0.001, effect size = 1.591) for the thorax onset latency, fast and moderate speeds (p = 0.003, effect size = 0.777) and fast and slow speeds (p < 0.001, effect size = 0.935) for the pelvis onset latency, fast and moderate speeds for the leading foot (p = 0.003, effect size = 0.878), fast and slow speed (p < 0.001, effect size = 0.913), showing that these were greater with an increase in turn speed, and moderate and slow speeds (p = 0.022, effect size = 0.325) for peak head yaw velocity, fast and slow speed (p = 0.027, effect size = 0.741) for peak head on pelvis, showing that these were less with an increase in turn speed. For step duration, a significant difference between fast and moderate speeds (p = 0.003, effect size = 0.856), fast and slow speeds (p < 0.001, effect size = 0.996) and moderate and slow speeds (p = 0.001, effect size = 0.823), showing that these were greater with an increase in turn speed (Table 3).

The comparison of TUG revealed by the Mann–Whitney *U* test found no significant difference between male and female (Table 4). In addition, we observed the strategy of turning 180° and asked about the speed of turning difficulty. The results show that there is no significant difference in all observations between males and females (Table 4).

**Table 4:** Comparison of timed up and go test (TUG), strategy used of turning 180° and speed difficulty.

Comparisons	Male (n = 25)	Female (n = 25)	p-value^#^	Effect size
Time up and go test (TUG)	14.38 (13.04, 16.05)	14.60 (13.66, 16.36)	0.357	0.287
Strategy used for turning 180° (n, %)				
Fast speed			0.490	–
Spinning	0 (0%)	2 (8%)		
Step	25 (100%)	23 (92%)		
Moderate speed			0.490	–
Spinning	0 (0%)	2 (8%)		
Step	25 (100%)	23 (92%)		
Slow speed			0.490	–
Spinning	0 (0%)	2 (8%)		
Step	25 (100%)	23 (92%)		
Speed difficulty (n, %)				
None	16 (64%)	15 (60%)	1.000	–
Fast speed	6 (24%)	7 (28%)		
Moderate speed	0 (0%)	0 (%)		
Slow speed	3 (12%)	3 (12%)		

## Discussion

This study aimed to explore turning characteristics in healthy young adults with a focus on differences in biological sex and the effects on body coordination and stepping characteristics when turning at different speeds. We expected there would be differences in whole-body coordination and stepping characteristics between the biological sexes with females having less coordination for the whole-body than males during turning.

The data showed that males and females had the same strategy used for turning 180° no matter how fast they turned. In addition, they used step duration and total number of steps to an equal extent regardless of difference in speeds as shown by non-significant differences in the step duration and total step. There were also no significant differences in other variables between the two groups. The only significant differences between the male and female groups were during fast turning for the leading foot onset, and in the slow speed for the total step. The similarity of turning kinematic characteristics in our population regardless of biological sex are consistent with a previous study, specifically following the top-down sequence as analysed from segment onset latencies^30^. Additionally, the segment onset latencies were similar to those in the younger adult group in the previous study^30^. Regarding the significant difference in the leading foot onset between male and female at fast speed, the raw data from the male group also showed a faster speed than the female group. This finding might reflect the response to the visual or auditory stimuli used in this study. The motor cortex and motor planning would control those responses, which are represented by the reaction time. Importantly, based on a previous study, males had faster responses in comparison to females^41^. However, it is important to note that generalizations about biological sex can vary depending on individual circumstances, and not all females or males may fit into these general observations.

When comparing between speeds within each group, the strategy of turning was found to be consistent with a previous study with regard to the top-down sequence and was dependent on the different speeds in both biological sexes^29^. There were, however, significant differences in head, thorax, pelvis, and leading foot onset latencies, peak head on pelvis and step duration with turn speed regardless of biological sex. These findings indicate that the difference in biological sex may not influence those variables.

These findings are pertinent to segment onset latency and are consistent with previous studies indicating that different speeds during turning may impact on whole-body coordination and stepping characteristics regardless of any differences in biological sex^29^. However, some characteristics including trailing foot onset latency, step size, and total number of steps may be influenced by biological sex which was highlighted by significant differences within the males only when comparing different turning speeds, with no such differences seen in the females. With regards to the step size, the result in the male group decreased, while the total step increased during slower turns, a finding consistent with a previous study. In contrast, the step size in the female group did not reduce during slower turns in comparison to the fast turns. When considering the step frequency, the results show a large IQR for this parameter when turning at slow speed compared to the other speeds. This implies that there is a wide variation in step frequency which could be due to various factors such as differences in balance, stride length, or overall and underlying health conditions^11,13–15^. In addition, participants within the female group showed a significant difference in peak head yaw velocity, which was not found in the male group. The pairwise comparisons found a significant difference among speeds within the female group between fast and slow speed, and moderate and slow speeds for peak head yaw velocity. The results in the female group are consistent with the previous study which found that peak head yaw velocity reduced during turning in a slow speed^29^. This finding is not consistent with the results in the male group because the variability of difference between the speeds did not reach significance.

Thomas et al.^42^ found that familiar biological sex differences such as those in anthropometry, flexibility, or strength could not explain the distinctive movement patterns used by females when compared to their male counterparts. They suggested a potential role for sociocultural constraints on the stereotypical movement patterns of females, whereby forward inclination of the trunk is reduced compared to that of males in tasks that necessitate some bending of the trunk. This suggests a potential role for sociocultural constraints on the stereotypical movement patterns of females^42^. Furthermore, it is essential to consider various factors that could contribute to this perception. For instance, females tend to have a lower centre of gravity compared to males due to differences in body composition^43–45^. This can affect their balance and stability control. Additionally, hormonal changes during the menstrual cycle can also affect a woman's balance^46,47^. Fluctuations in hormone levels can also lead to changes in joint laxity and muscle coordination, potentially impacting the ability to maintain balance^47^.

In addition to whole-body coordination and step characteristics, this study found a non-significant difference in the TUG test results in relation to clinical outcome for dynamic balance, which did not support our hypotheses. Generally, females took longer to complete the TUG test in comparison to males, which is consistent with previous studies^25,48,49^. These disparities may be attributed to the female participants being generally shorter, and of lower weight, and also due to accelerated decline in muscle mass after the age of 55, leading to shorter stride length and reduced muscle strength^16,50^. Consequently, this factor may have influenced the outcomes of our study, where a significant difference in TUG within the specific age range examined was not observed. This suggests that biological sex may play a more prominent role in the performance of older adults than the specific age group investigated in our study.

The study into differences in biological sex with regards to turning needs to be expanded beyond biological factors to include environmental and sociocultural factors that influence motor skills. To investigate differences between females and males in healthy young adults, it may also be fruitful to consider participants on an individual basis, taking account of their skills, histories, strength, anatomical differences and cultural attitudes.

There are several limitations to this study. Firstly, several of the characteristics of the participants, including body weight and height, were not controlled. This lack of control of these variables may have had an impact on whole-body coordination and stepping characteristics. Previous evidence has indicated that there is a relationship between individuals who are overweight and obese and an increased risk of falls, as individuals with a higher centre of gravity and more pronounced lumbar lordosis are more prone to falling^51^. Secondly, as a result of the variations in body weight and height, the risk of balance loss and falls varies between the biological sexes. For instance, female with obesity have been found to have a greater risk of recurrent falls, whereas male that are underweight are associated with a higher risk of falls^51^. With regard to the transferability of the conclusions, as this study was conducted in Thailand the findings may not be directly relevant to populations in other countries due to the differences in average weight and BMI due to genetic, lifestyle, and cultural variations. Thirdly, the age range of participants was wide, particularly in the female group, having a significantly broader age range in comparison to the male group. However, no statistically significant difference was found between the two groups, and the mean ages were similar. Fourthly, this sample had an average age of 20 years old which may not show the differences in physiology and other factors between males and females which may be seen in older adults. Finally, we did not investigate the hormonal factors that can influence both physical performance and mental health of both groups. Our study suggested that future research in this field could set criteria for participant selection including maximum level of education, category of employment, current monthly salary, etc. Cultural attitudes could be assessed by questions including use of leisure time (e.g. exercise regimes, dining out, dietary focus), place of habitation (with parents etc.), and caring responsibilities. Additionally, any further study could consider factors associated with hormones for both groups and the menstrual cycle when investigating female participants.

## Conclusion

This study has shown that there is a potential influence of biological sex, especially in females, on the leading foot onset during fast speed turning and the total number of steps during slow speed turning. Additionally, different speeds can affect multiple variables in both males and females, with females appearing to experience more difficulties in whole-body coordination during turning compared to males. However, it is important to note that these biological sex differences in turning at different speeds did not have a significant impact on the risk of falling, as indicated by the non-significant difference in TUG. Thus, it is crucial to consider individual differences rather than making generalisations. Turning is a complex attribute that can be influenced by various factors. Our study has shown that biological sex is one factor that could affect whole-body coordination and stepping characteristics during 180° standing turns.

## Data Availability

The data presented in this study are available on request from the corresponding author.
